# The Protective Effect of Radix Polygoni Multiflori on Diabetic Encephalopathy via Regulating Myosin Light Chain Kinase Expression

**DOI:** 10.1155/2015/484721

**Published:** 2015-06-25

**Authors:** Yu He, Feng Wang, Shiqiang Chen, Mi Liu, Wei Pan, Xing Li

**Affiliations:** Guizhou Medical University, Affiliated Hospital of Guizhou Medical University, Guiyang, Guizhou 550025, China

## Abstract

Currently there has been no effective treatment of diabetic encephalopathy. Radix Polygoni Multiflori, a famous traditional Chinese medicine, is widely used in antiaging treatment, especially in prevention and treatment of Alzheimer's diseases. In this study we tried to explore the effect of Radix Polygoni Multiflori on cognitive function among diabetic rats with demonstrated cognitive impairment. SD rats were divided into group A (control group), group B (diabetes), group C (treated with Radix Polygoni Multiflori at the dose of 2 g/kg/d), and group D (treated with same drug at the dose of 1 g/kg/d). The results showed that 8 weeks of Radix Polygoni Multiflori treatment could improve the cognitive dysfunction of diabetic rats (*P* < 0.01), recover the ultrastructure of hippocampal neurons, and increase the number of synapses in a dose-dependent manner. Further experiment also suggested that the neuroprotective effect of Radix Polygoni Multiflori was partly achieved by downregulating MLCK expression in hippocampus via ERK signaling.

## 1. Introduction

Diabetic encephalopathy is widely recognized as one of the chronic complications of diabetes. Metabolic disorder complicated by diabetes could lead to central nervous system damage. The associated cognitive dysfunction as well as physiological and structural changes in the cerebral nerve will ultimately result in diabetic encephalopathy [1, 2]. Present studies suggest that central nervous system damage is related to metabolism disorders of protein and lipid that are caused by high glucose. Metabolic abnormalities destroy the homeostasis of calcium in neurons, leading to neurodegenerative changes and irreversible neuronal apoptosis [3]. Myosin light chain kinase (MLCK) is an important kinase involved in the regulation of calcium pathway. MLCK phosphorylates myosin light chain (MLC), which thereby increases the activity of myosin ATPas and starts the interaction between myosin and actin. This causes rearrangement of F-actin cytoskeleton, which ultimately results in cell shrinkage and the permeability change of cell [4]. MLCK is closely related to neurodevelopmental guiding factor and myosin dynamics. In neurons, MLCK can promote the recruitment of synaptic vesicles, enhance the release of neurotransmitters, and regulate the elongation, migration, and projections of growth axon [5–8]. In our previous work [9], by protein two-dimensional electrophoresis and mass spectrometry, we found the elevated expression of MLCK in hippocampal neurons of diabetic rat with cognitive dysfunction. And cognitive dysfunction was more severe in diabetic rats with extended duration of diabetic encephalopathy. MLCK expression increased in these rats, suggesting its potential involvement in the pathogenesis and development of diabetic encephalopathy.

There has been no effective treatment for diabetic encephalopathy till now. Radix Polygoni Multiflori is a famous tonic Chinese medicine, which is widely used in antiaging treatment and research about learning and memory, especially in prevention and treatment of Alzheimer's diseases in which cognitive impairment appears as in diabetes encephalopathy. Whether Radix Polygoni Multiflori can improve the cognitive function of diabetic encephalopathy remains unknown. In this study we explored the effect of Radix Polygoni Multiflori on cognitive function among diabetic rats with demonstrated cognitive impairment and tried to provide preliminary evidence to support the use of Radix Polygoni Multiflori in the prevention and treatment of diabetic encephalopathy.

## 2. Materials and Methods 

### 2.1. Animals and Ethics Statement

Male SD rats aged 4-5 weeks were obtained from Guiyang Medical Animal Center (Certificate of Conformity: scxk (Qian) 2002-0001). Rats were kept in groups of four in constant temperature (21 ± 2°C) until animal experiments. Food [CRM (P) Rat and Mouse Breeder and Grower, standard pelleted diet (Special Diet Services, Guiyang College, China)] and water were provided ad libitum. All experimental procedures were performed under strict adherence to regulations and in accordance with the Animals (Scientific Procedures) Act. The animal experiments were licensed and approved by the local ethical review committee.

### 2.2. Preparation of Radix Polygoni Multiflori Extract

Radix Polygoni Multiflori (purchased from Guizhou Tongjitang Pharmacy) was cut into pieces and then boiled with fresh water for three times (each time for 30 minutes). The decoction from each time of boiling was collected and mixed. The decoctions were then heated until the volume decreased to two thirds of the original. The liquid was setting to cool down. 75% ethanol of one- to twofold volume was slowly added to the liquid and stirred slowly. Then the liquid was sealed and refrigerated for 24–48 h and filtered, and the solution was placed in a rotary evaporator under reduced pressure and cold temperature to recover ethanol. Recycling solution was poured into the evaporating dish and then was placed at a constant temperature of 100°C water bath evaporation to extract, cold standby, diluted to a certain concentration aside. Clinical medication standard of Radix Polygoni Multiflori was 6~12 g according to Chinese Pharmacopoeia 2010 edition. According to the experimental dose translation (translated equivalent dose conversion coefficient method), the conversion factor should be 6.3. Assuming average body weight for Chinese adult is 60 kg; then dose for rats = (6~12) g/60 kg × 6.3 = (0.63~1.26) g/kg. Set up two dose groups: high-dose Radix Polygoni Multiflorigroup (2 g/kg), low dose Radix Polygoni Multiflori group (1 g/kg).

### 2.3. Establishment of Diabetic Rat Model

Rats were fed with adaptive diets in lab for one week and the cognitive function was normal determined by Morris water maze, also the fasting blood glucose value was normal with 3.0–5.0 mmol/L; 48 male SD rats of 180–220 g were divided into four group: (1) group A (control group) (intraperitoneal injection of citric acid-sodium citrate buffer (0.1 mol/L, pH 4.2)); (2) group B (T1DM group): the animals were fasted 12 hours before intraperitoneal injection of streptozotocin STZ (50 mg/kg, Sigma), the fasting blood glucose ≥ 16.7 mmol/L after 72 hours, and fasting blood glucose was maintained at 16.7 mmol/L or more for 3 weeks; (3) group C (high dose of Radix Polygoni Multiflori group), four weeks after T1DM, rats were tube-fed with Radix Polygoni Multiflori (2 g/kg/d) for 12 weeks; (4) group D (low dose of Radix Polygoni Multiflori group): rats were also tube-fed for 12 weeks, however the dose of Radix Polygoni Multiflori was 1 g/kg/d. Rats in groups A and B were tube-fed with the same volume of saline for 12 weeks. 12 weeks later, the Morris water maze was conducted to test cognitive function of rats again, after which the rats were decapitated and the hippocampus was isolated and stored at −80°C.

### 2.4. Morris Water Maze Test

Morris water maze test (developed by Institute of Materia Medica, Chinese Academy of Medical Sciences) includes (1) navigation test: The day before the experiment all the rats are put into the water to swim freely for 2 min, to recognize maze environment. Experiment lasted five days; each day is divided into two time periods (morning and afternoon). Four times of training were given during each period. In total, eight times of training were given per day. When training begins, the platform was placed in the fourth quadrant and the rats facing the wall from the midpoint of the four quadrants of the wall are put into the pool, recording their time to find the hidden underwater platform (escape latency). The rats were trained four times from different quadrants into the water, the ones which could not find the platform in 60 seconds would be escorted to the platform, and the escape latency was denoted as 60 s, each rat was allowed to rest for 30 seconds on the platform. The escape latency was recorded at the last day of the experiment and the mean was calculated. (2) Space exploration experiment: the space exploration experiments were performed at the afternoon of the fifth day, the platform should be removed before the experiment, one quadrant should be chosen and the rat should be put into the pool, the swimming track is recorded in 60 s, the memory of rats is examined to the platform. The first-passage time of the target area and the times of the cross of the location of the platform in 60 s were recorded.

### 2.5. Ultrastructural Changes Examination of the Hippocampal Neurons Using Electron Microscopy

The hippocampal tissue was fixed in the electron microscopy fixing buffer, making ultrathin tissue sections, transmission electron microscopy (Hitachi H-7650) was used to observe the neuron ultrastructure.

### 2.6. The Expression of MLCK and NMDA Receptor in Hippocampus by Double Immunofluorescence Staining

Hippocampus tissues from rats in four groups were embedded in paraffin after fixing with 10% paraformaldehyde. Hippocampus sections were deparaffinized and incubated with 3% hydrogen peroxide for 20 minutes to eliminate endogenous peroxidase activity. Hippocampus tissue samples were washed three times by PBS. Immunnohistochemical staining was enhanced under microwave antigen retrieval and antigen was blocked with normal goat serum solution at 37°C for 20 min. Cy3-MLCK murine monoclonal antibody and FITC-NMDAR2B murine monoclonal antibody (Biosynthesis Biotechnology) at a dilution of 1 : 100 were dropped and incubated for 45 min at 37°C. PBS was washed 3 times and the slides were mounted by antiquenching fluorescence sealed tablets and photographed under a fluorescence microscope.

### 2.7. Detection of the Protein Level of MLCK, Erk1/2, and pErk1/2 in Hippocampus by Western Blot

The cell lysates extracted from the hippocampus tissues of mice were used for immunoblot analysis. Equal amounts of protein of 35 *μ*g from each sample were subjected to 10% or 5% SDS PAGE gels electrophoresis, and protein was transferred to PVDF membranes; subsequently, the membranes were blocked. After that, the membranes were incubated with mouse anti-MLCK monoclonal antibody (1 : 1000, sigma), rabbit anti-Erk monoclonal antibody (1 : 500, Cell Signaling Technology), rabbit anti-pErk monoclonal antibody (1 : 1000, Cell Signaling Technology), and mouse anti-*β*-actin monoclonal antibody (1 : 400, Beijing Zhongshan Golden Bridge) at 4°C overnight. Then the PVDF membranes were incubated with the horse-radish peroxidase-conjugated secondary antibody goat anti-mouse or goat anti-rabbit lgG secondary antibodies (1 : 20000). The protein bands were analyzed in ECL chemiluminescence system (Thermo).

### 2.8. Statistics Analysis

Data are expressed as mean ± standard deviation (±s) and according to the One-way ANOVA and Pearson correlation analysis in the statistical software SPSS19.0, *P* < 0.05 was considered statistically significant difference.

## 3. Results

### 3.1. The Expression of MLCK Was Elevated in Hippocampal Tissue in Diabetic Rats and Coexpressed with NMDA Receptor

**Figure 1:**
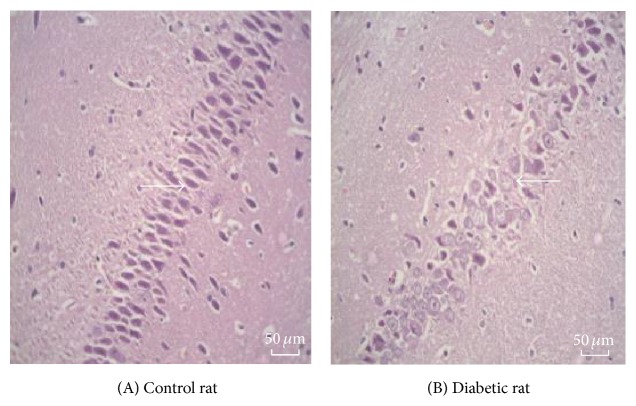
The representative photos of hippocampal tissue in rats. (A) Control rat; (B) diabetic rat. The neurons in rat hippocampal tissue (mainly pyramidal cells) in control rat group are round or oval in shape, with large and lightly stained cytoplasm also round in shape and orderly arranged nucleus. In diabetes rat group the morphology of the neurons was obviously abnormal in hippocampal tissue. The number of neurons decreased, cells were swelling, and nuclear fragmentation or disappearance was observed, as in cell apoptosis.

The neurons in rat hippocampal tissue (mainly pyramidal cells) in group A showed normal morphology that is round or oval in shape, with large and lightly stained cytoplasm also round in shape and orderly arranged nucleus. Compared with that of group A, the morphology of the neurons was obviously abnormal in hippocampal tissue in group B. The number of neurons decreased, cells were swelling, and nuclear fragmentation or disappearance was observed, as in cell apoptosis (Figure 1).

**Figure 2:**
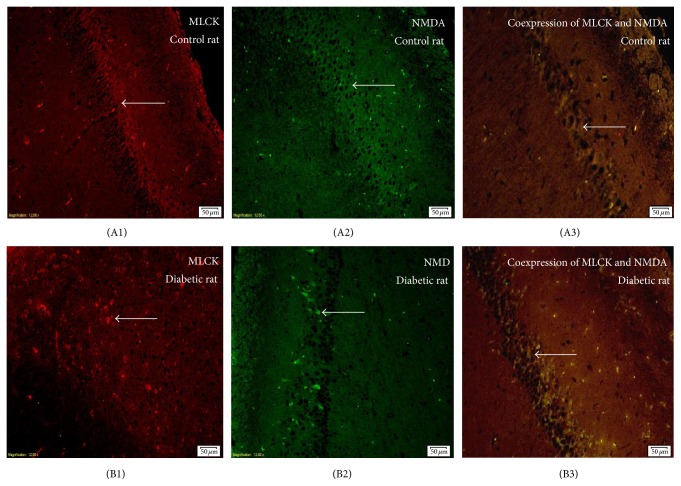
The representative photos of MLCK (red light) and NMDAR2B (green light) expressions in hippocampal tissue in rats by immunofluorescence staining. The coexpression of MLCK and NMDAR2B was shown as yellow light. (A1)–(A3), control rats; (B1)–(B3), diabetic rats. Immunofluorescence staining showed that the expression of MLCK and the NMDA receptor and their coexpression in the rats hippocampus in diabetic rats (group B) were significantly higher than rats in control rats (group A) (*P* < 0.05) (Figure 2, Supplementary Table 1). According to the Person correlation analysis, the expression of MLCK and NMDAR2B showed a positive correlation (*r* = 0.958, *P* < 0.01) (Supplementary Figure 1).

Immunofluorescence staining showed that the expression of MLCK and the NMDA receptor in the rats hippocampus in group B was significantly higher than rats in group A. Coexpression of MLCK and NMDA receptor was also significantly higher than that in group A, which showed a statistically significant difference (*P* < 0.05) (Figure 2, Supplementary Table 1 in Supplementary Material available online at http://dx.doi.org/10.1155/2015/484721, the expression of MLCK showed in red fluorescence, the expression of NMDAR2B in green fluorescence, and the coexpression in yellow fluorescence.). According to the Pearson correlation analysis, the expression of MLCK and NMDAR2B showed a positive correlation (*r* = 0.958, *P* < 0.01) (Supplementary Figure 1).

### 3.2. Radix Polygoni Multiflori Could Improve Cognitive Function in Diabetic Rats

After 12 weeks of animal model establishment, in Morris water maze test, compared with rats in group A, rats in group B had significantly prolonged escape latency and first-passage time, which significantly reduced the number of crossing (*P* < 0.01). After intervention with different doses of Radix Polygoni Multiflori, escape latency of rats in groups C and D were significantly reduced. Comparing with group B, these two groups with intervention also had significantly less first-passage time and significantly higher crossing frequency, and the effect of Radix Polygoni Multiflori was dose-dependent (all *P* < 0.01, Supplementary Table 2).

### 3.3. Radix Polygoni Multiflori Induced Morphological Change in the Hippocampal Tissue of Diabetic Rat

**Figure 3:**
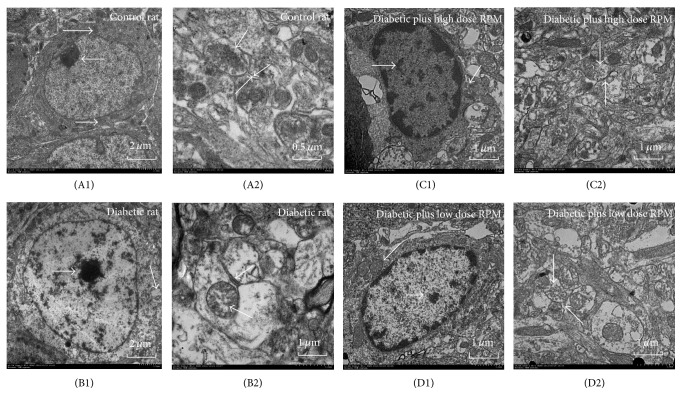
The ultrastructure of neuronal cells (A1, B1, C1, and D1) and synapses (A2, B2, C2, and D2) of hippocampal tissue in rats of each group. A: control rat; B: diabetic rat; C: diabetic plus high dose RPM; D: diabetic plus low dose RPM. Control rats showed regular nuclear morphology (Figure 3(A1)), large number of synapses in complete form, and rich synaptic vesicles (Figure 3(A2)). Nuclear membrane shrinkage was observed in diabetic rats and cell membrane structure was also unclear (Figure 3(B1)). Figure 3(B2) showed significantly reduced number of synapses, abnormal synaptic structure, and blur or missing synaptic cleft. Rat neuronal cell structure in diabetic plus RPM had been greatly improved compared with diabetic rats, in which diabetic plus high dose RPM was significantly higher than of in low dose RPM (Figures 3(C1), 3(D1) 3(C2), and 3(D2)).

Transmission electronic microscopy found that the hippocampal neurons of rats in group A showed regular nuclear morphology, where nuclear chromatin was rich and evenly distributed with prominent nucleoli and organelles were abundant in the cytoplasm including evenly distributed endoplasmic reticulum and tightly packed mitochondria (Figure 3(A1)). Large number of synapses in complete form and abundant synaptic vesicles were observed (Figure 3(A2)). The hippocampal neurons of rats in group B showed nuclear membrane shrinkage, with unevenly distributed nuclear chromatin, unclear cell membrane, irregularly distributed endoplasmic reticulum, and swollen mitochondria in irregular shape (Figure 3(B1)). In addition, the numbers of synapses and synaptic vesicles were significantly reduced, synaptic structure was interrupted, postsynaptic membrane was swelling, and synaptic cleft was blurred or even disappeared (Figure 3(B2)).

After Radix Polygoni Multiflori treatment, neuron cell structure in groups C and D had been greatly improved compared with group B. In particular, the improvement was more significant in group C than in group D. It was found that neuron cells treated with Radix Polygoni Multiflori presented relatively intact morphology, plenty of cytoplasmic organelles, rough endoplasmic reticulum and mitochondria, intact nuclear membrane, and uniform chromatin (Figures 3(C1) and 3(D1)). Notably, the number of synapses of neuron cells increased significantly in group C, and the distribution density of synaptic vesicles increased. There was no swelling around the postsynaptic membrane. The synaptic structure was complete and morphology was closed to group A (Figures 3(C2) and 3(D2)).

### 3.4. Radix Polygoni Multiflori Regulated the Expression of MLCK and ERK1/2

**Figure 4:**
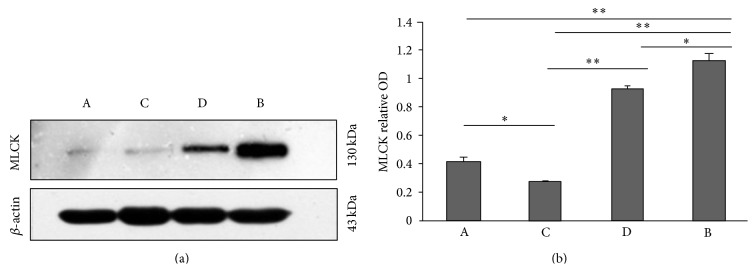
Western blot showed the expression of MLCK in hippocampal tissue in rats. (a) The representative photos of western blot. (b) The statistical analysis of western blot. Data shown are mean ± SEM. ^*∗*^ *P* < 0.05, ^*∗∗*^ *P* < 0.01.

Western blot showed that, compared with group A, the expression of MLCK in the hippocampal tissue of rats in group B was significantly elevated (*P* < 0.01). After Radix Polygoni Multiflori treatment, compared with group B, the expression of MLCK in rat hippocampus was significantly reduced. The decrease in MLCK expression in group C was more significant (*P* < 0.01) than that in group D, suggesting a dose-dependent relationship. Meanwhile, the expression of MLCK in the rat hippocampus in group C was slightly lower than that in group A (*P* < 0.05, Figure 4).

**Figure 5:**
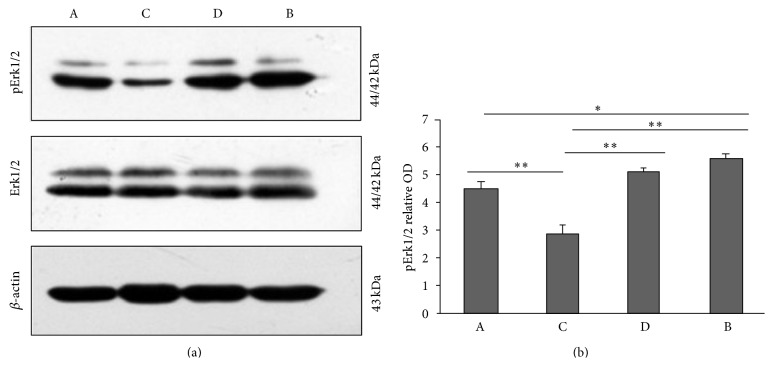
Western blot showed the expression of Erk1/2 and pErk1/2 in hippocampal tissue in rats. (a) The representative photos of western blot. (b) The statistical analysis of western blot. Data shown are mean ± SEM. ^*∗*^ *P* < 0.05, ^*∗∗*^ *P* < 0.01.

Compared with group A, the phosphorylation level of ERK in the rat hippocampus in group B was elevated (*P* < 0.05). After Radix Polygoni Multiflori treatment, compared with group B, the phosphorylation level of ERK in the rat hippocampus in group B was significantly reduced. Group C has more significant reduction in ERK phosphorylation in comparison to group D, which also suggests a possible dose-dependent relationship (*P* < 0.05). Meanwhile, the phosphorylation level of ERK in the rat hippocampal tissue in group C was slightly lower than that in group A (*P* < 0.01, Figure 5). The phosphorylation level of ERK showed a positive correlation with the expression of MLCK (*r* = 0.799, *P* < 0.01, Supplementary Figure 2).

## 4. Discussion

Radix Polygoni Multiflori is one of the most precious Chinese herbal medicines. It has reputation of benefiting liver and increasing stamina. Many thousand years of practice of clinical practice traditional Chinese medicine has demonstrated the effect of Radix Polygoni Multiflori in terms of preventing dementia and improving memory. It has also been considered effective in antiaging and increasing longevity. According to modern medicine, the effects of Radix Polygoni Multiflori include enhancing immunity, antioxidation, increasing DNA repair, and decreasing LDL-Cholesterol and improving adipose metabolism.

The cell proliferation, migration, differentiation disorders, apoptosis, and nerve regeneration dysfunction of hippocampal neuron play an important role in the development of diabetic encephalopathy. Magariños and McEwen [10] demonstrated that this change around the hippocampal synapses of diabetic rat indicated that the changes in synaptic plasticity of the hippocampus were involved in the development and progression of diabetic cognitive dysfunction. In this study, we demonstrated that the dysfunction of spatial learning and memory in diabetic rats was accompanied by a change in the number and structure of hippocampal neurons and synapses. Radix Polygoni Multiflori plays a role in improving memory and fatigue and has other neuroprotective effects [11]. Therefore, it is reasonable to speculate that Radix Polygoni Multiflori might prevent or delay the neuronal degeneration and necrosis, protect synapse structure, improve the effectiveness of synaptic transmission, and function in the prevention and treatment of diabetes associated cognitive impairment.

Hippocampus is the key part of learning and memory in the brain. Long-term potentiation (LTP), the phenomena of enhanced synaptic plasticity of the hippocampus, is the molecular basis of learning and memory. The receptor of ion channel N-methyl-D-aspartate (NMDA) is essential in the formation and maintenance of LTP [12]. The main mechanism of NMDA receptor that induced learning and memory impairment is via the excitotoxicity of NMDA receptor. The abnormal release of the neurotransmitter glutamate around the cells promotes the opening of NMDA receptor channel and results in the inflow of calcium. This affects the downstream reactions and gene transcriptions, which leads to the pathological LTP in the hippocampus region, causing learning and memory impairment [13]. We are interested in finding out, as a membrane receptor, whether NMDA expression is related to cytoskeletal proteins and whether it is regulated by MLCK. Lei et al. [14] found that MLCK was expressed in the cell body and dendrites of neurons in the primary cultured rat hippocampal CA1 region. After cells were treated with MLCK inhibitors (ML-7 or AV25), NMDA-mediated excitatory postsynaptic microcurrent (mEPSCNMDA) was decreased; after cells were treated with MLCK constitutive activator, mEPSCNMDA was enhanced. These results indicated MLCK could regulate the function of NMDA receptor. Our study demonstrated that, in the diabetic encephalopathy rats, expression of MLCK and NMDA receptors and their coexpression in hippocampus were both significantly increased. The positive correlation found between the expression of MLCK and NMDA receptors further supported that in diabetic encephalopathy MLCK might regulate the expression of NMDA receptors and then participated in the development of learning and memory impairment.

MLCK is not only involved in the process of the contraction, movement, migration, and apoptosis of smooth muscle cells [15, 16], but also plays an important role in the nonsmooth muscle cells. This study and the preliminary studies [9] showed that the expression of MLCK was significantly increased in the hippocampal neurons of the diabetic encephalopathy rats, suggesting that MLCK might be involved in the development of diabetic encephalopathy. After Radix Polygoni Multiflori intervention, the cognitive impairment in diabetic encephalopathy rats was improved while the expression of MLCK in the hippocampus was significantly decreased, indicating that Radix Polygoni Multiflori might play a role in downregulating the expression of MLCK in the hippocampus through some unknown mechanism. It is demonstrated that tetrahydroxystilbene-2-O-*β*-D-glycoside (TSG), the unique biological active component in Radix Polygoni Multiflori, is a soluble antioxidant, with effect of free radical scavenging, neuronurturing, and other neuroprotective effects. It can improve the survival rate of damaged nerve cells, reduce the lactate dehydrogenase leakage from the cells, and inhibit glutamate-induced calcium overload in the cell. Among rats with diabetic encephalopathy, the downregulation of MLCK expression in hippocampus appeared after Radix Polygoni Multiflori treatment. Whether this phenomenon is associated with TSG is a further question yet to be answered.

It is demonstrated that MLCK is at the downstream of ERK in the regulation of myosin dynamics. It is observed that, in the LM-MCF-7 cells, MLCK expression and the phosphorylation level of its downstream factor MLC were significantly inhibited after ERK inhibition. Our results showed that Radix Polygoni Multiflori could reduce the expression of MLCK in hippocampal tissue of diabetic rats; simultaneously the phosphorylation level of ERK was significantly decreased. The trend between them was consistent and had a positive correlation (*r* = 0.799, *P* < 0.01). In the group treated with high doses of Radix Polygoni Multiflori, the expression of MLCK in the hippocampus and the phosphorylation level of ERK were lower than control group and the trend was consistent and had a positive correlation, indicating that the effect of Radix Polygoni Multiflori on MLCK expression and ERK phosphorylation were both dose-dependent.

In conclusion, our study showed that MLCK might be involved in the development and progression of diabetic encephalopathy. Its regulatory effect to NMDA receptors might be one of the underlying mechanisms. The protective effect of Radix Polygoni Multiflori against diabetic encephalopathy might be associated with hippocampus ERK/MAPK signal transduction pathway and MLCK expression. The cognitive impairment related by diabetes is therefore improved by Radix Polygoni Multiflori. This study might benefit us to understand the pathogenesis of diabetic encephalopathy and provide a candidate for the diagnosis and detection of diabetic encephalopathy, as well as a potential target for drug discovery.

## Supplementary Material

Optical density levels for MLCK and NMDAR2B were both significantly higher in Group B.
