# Supplementary material for: The Protective Effect of Radix Polygoni Multiflori on Diabetic Encephalopathy via Regulating Myosin Light Chain Kinase Expression
Source: J Diabetes Res. 2015 Jun 25;2015:484721. doi: 10.1155/2015/484721 (PMC4496489; doi:10.1155/2015/484721)
Supplement: Supplementary file 1 — Optical density levels for MLCK and NMDAR2B were both significantly higher in Group B. [file 484721.f1.pdf]

Supplementary Figure legends

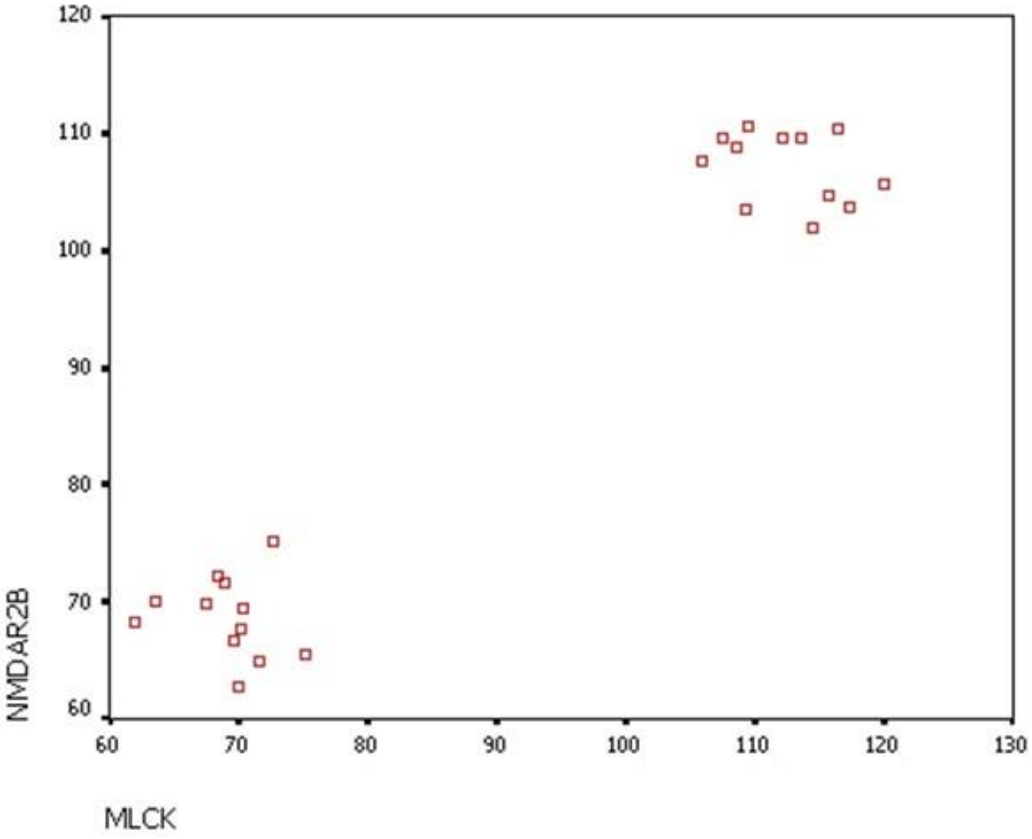

Supplementary FIGURE.1 The Correlation analysis of MLCK and NMDAR2B expression（r=0.958，P < 0.01）

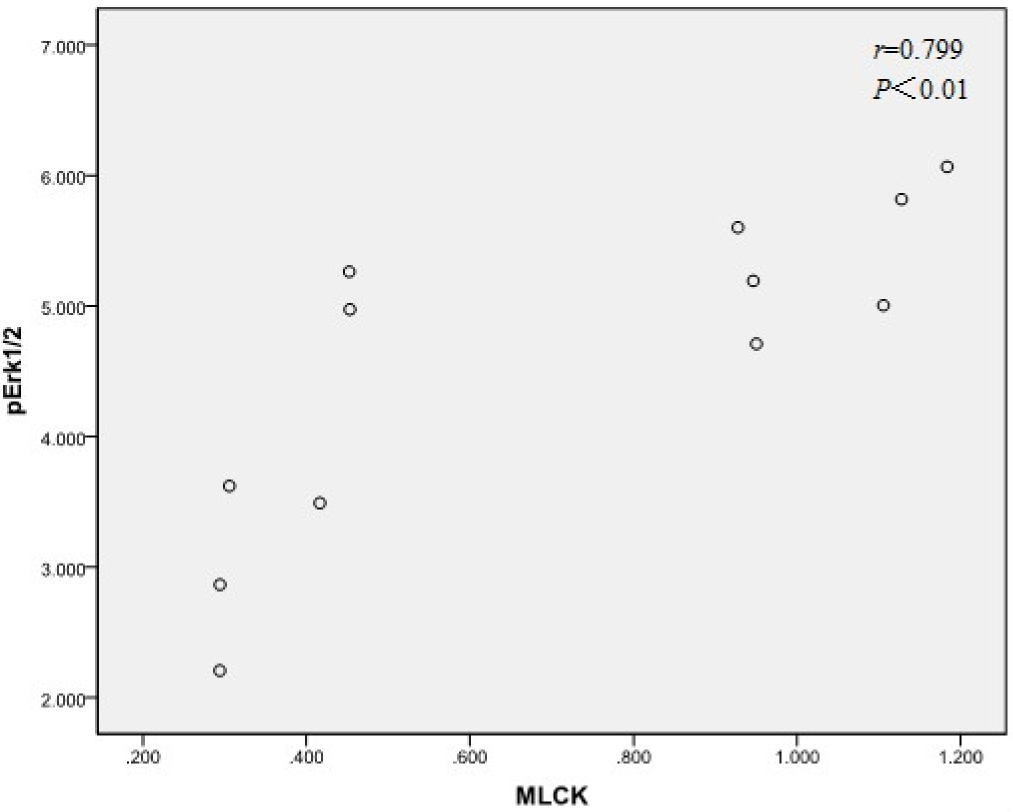

Supplementary FIGURE. 2　The Correlation analysis of MLCK and phosphorylated ERK expression

（r=0.799,P＜0.01）

Supplementary TABLE1 Optical density values of MLCK and NMDAR2B in hippocampal tissue in rats by immunofluorescence staining

	Group A（n=20）	Group B（n=31)
MLCK	69.31±9.36	112.31±8.73*
NMDAR2B	68.92±8.33	106.91±8.44*
Co-expression of MLCK andNMDAR2B	61.33±8.55	101.82±7.68*

* P<0.05, compared to group A

Supplementary Table2 The results of morris water maze test

	Group A （n=12）	Group B （n=12）	Group C （n=12）	Group D （n=12）
escape latency（s）	12.97±7.98	30.40±12.68$^{\triangle}$	14.73±7.49**	18.23±6.56*
first-passsge time（s）	8.43±7.35	32.77±19.66$^{\triangle}$	10.58±6.53**	16.29±8.74*
The times of cross	3.62±2.31	1.44±1.34$^{\triangle}$	3.60±2.12**	2.25±1.59*

△compared with group A, *P*<0.01; * compared with group B, *P*<0.05，** compared with group B, *P*<0.01
